# Lysophosphatidic acid (LPA)-antibody (504B3) engagement detected by interferometry identifies off-target binding

**DOI:** 10.1186/s12944-021-01454-4

**Published:** 2021-04-14

**Authors:** Manisha Ray, Yasuyuki Kihara, Darryl J. Bornhop, Jerold Chun

**Affiliations:** 1 Translational Neuroscience Program, Sanford Burnham Prebys Medical Discovery Institute, La Jolla, CA 92037 USA; 2 Department of Chemistry and Vanderbilt Institute for Chemical Biology, Nashville, TN 37235 USA

**Keywords:** Lysophosphatidic acid, Compensated interferometric reader, Ligand binding, Antibody, Lpathomab, Lysophospholipid

## Abstract

**Background:**

Lysophosphatidic acid (**LPA**) is a bioactive lysophospholipid that acts through its six cognate G protein-coupled receptors. As a family, lysophospholipids have already produced medicines (e.g., sphingosine 1-phosphate) as is being pursued for LPA through the use of specific antibodies that reduce ligand availability.

**Methods:**

The binding properties of a commercially available, reportedly specific, monoclonal LPA antibody named **504B3** that is related to the clinical candidate Lpathomab/LT3015 were reexamined using a free solution assay (**FSA**) measured in a compensated interferometric reader (**CIR**).

**Results:**

Measurement of 504B3 binding properties with an FSA-CIR approach revealed similar binding affinities for 504B3 against LPA as well as the non-LPA lipids, phosphatidic acid (**PA**) and lysophosphatidylcholine (**LPC**).

**Conclusions:**

Antibody binding specificity and sensitivity, particularly involving lipid ligands, can be assessed in solution and without labels using FSA-CIR. These findings could affect interpretations of both current and past basic and clinical studies employing 504B3 and related anti-LPA antibodies.

## Introduction

Lysophosphatidic acid (**LPA**) is a potent, bioactive lipid that acts through six cognate G protein coupled receptors (**GPCRs**) named LPA_1–6_ [1, 2]. LPA signaling is involved in many physiological processes such as cell proliferation, chemotaxis, and smooth muscle contraction, as well as cell survival [3]. Elevated LPA levels are associated with multiple disease pathologies including cancer, hydrocephalus, and fibrosis [3–6], implicating the therapeutic potential of modulating LPA pathways to reduce cognate receptor activity. This strategy has already generated four medicines through the related lysophospholipid, sphingosine 1-phosphate (**S1P**), whose receptor modulators are being used in the treatment of multiple sclerosis (fingolimod, siponimod, ozanimod and ponesimod) [7].

A complementary strategy that reduces ligand availability is being pursued through the use of specific antibodies to lower LPA levels [8–12]. In 2011, Lpath Inc. (merged with Apollo Endosurgery, Inc. in 2016) developed humanized monoclonal anti-LPA antibodies, including a phase 1a molecule, Lpathomab/LT3015 [10, 12]. The binding affinity and selectivity of Lpathomab/LT3015 was previously determined by enzyme-linked immunosorbent assay (**ELISA**) using unnatural, biotinylated 18:0 LPA [13], which showed highly specific and strong binding affinity to 18:1 LPA, without reported binding to other lipid species, including S1P, 18:0-lysophosphatidylcholine (**LPC**), phosphatidic acid (**PA**), phosphatidylcholine (**PC**), and platelet-activating factor (**PAF**).

A related, derivative anti-LPA antibody, 504B3, whose complementarity determining regions show 79% identity to Lpathomab/LT3015 [11], is commercially available (Echelon Biosciences, Product Number: Z-P200). ELISA studies have reported similar specificity and selectivity of 504B3 compared to Lpathomab/LT3015 [8]. Additional in vitro studies using Kinetic Exclusion Assays (KinExA) demonstrated a high affinity (× 4 times higher than LPA receptors) of 504B3 for all relevant LPA species [14], particularly 16:0 and 18:2 LPA that are abundant in the CSF of injured mice and humans, suggesting uses in the central nervous system following neurotrauma, and in inflammatory sites (tumor cell growth, rheumatoid arthritis). The reported binding data on specificity of 504B3 stimulated its use for preclinical studies of spinal cord [8] and traumatic brain injuries [9, 14].

ELISA is a commonly used assay for determining binding affinity but can possess a number of technical drawbacks that may impact the accuracy of measuring ligand specificity. These include multiple washing steps that may under or overestimate affinities, immobilization that can increase non-specific interactions [15, 16], and particularly, the use of biotinylated lipids that limit conformational flexibility of the lipids and introduce non-native epitopes [13].

An alternative methodology is interferometry, particularly interferometric assays that use unmodified, native binding partners in a free solution to measure the light refractive index change that occurs as a result of binding-induced conformational and/or hydration changes produced by real-time binding events in a *sample* compared to a non-binding *reference* [17–20]. A first generation technique, back scattering interferometry (**BSI**) [21], demonstrated the use of these free solution assays in determining antibody binding against native neuroactive molecules (such as serotonin, histamine, and dopamine) [22]. More recently, a compensated interferometric reader (**CIR**: 2nd generation BSI) was used to measure the native equilibrium binding (K_D_s) between different LPA forms and one of its receptors, LPA_1_, in the low nanomolar range [18, 19], demonstrating the high sensitivity and specificity detection capabilities of the CIR. This report further demonstrates the versatility of a label-free, free solution assay (**FSA**) in combination with CIR (**FSA-CIR**) by measuring the specific binding of LPA and non-LPA lipid ligands to the anti-LPA antibody, 504B3, in a more native environment.

## Materials and methods

### Lipid sample preparation

**Fig. 1:**
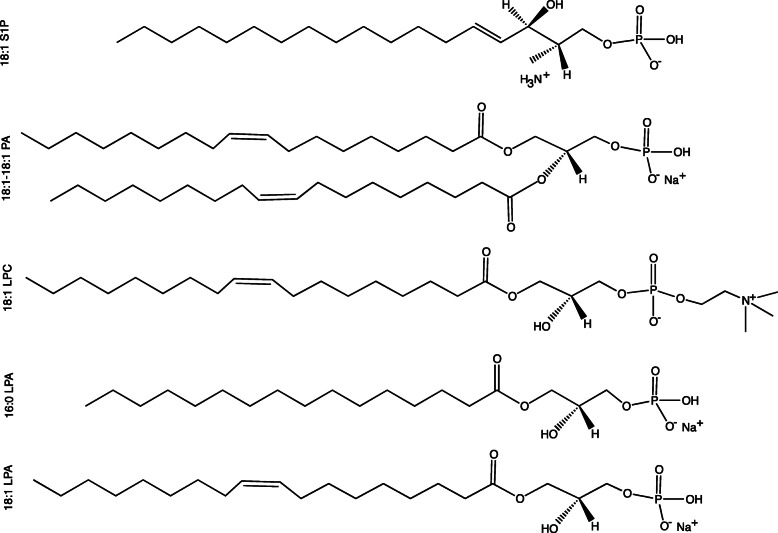
Chemical structure of ligands used in the binding assays against the anti-LPA antibody, 504B3. Lipids varied by acyl chain length (16:0, 18:1 LPA), head group (18:1 S1P, 18:1 LPC), or the presence of a 2nd acyl chain (18:1–18:1 PA)

The murine antibody, 504B3, was purchased from Echelon Biosciences (Salt Lake City, UT, USA). All lipid species including, 1-oleoyl-LPA (18:1), 1-palmitoyl-LPA (16:0), 1-oleoyl-lysophosphatidylcholine (18:1 LPC), 1,2-dioleoyl-PA (18:1–18:1), and sphingosine 1-phosphate (d18:1) (Fig. 1) were purchased from Avanti Polar Lipids Inc. (Alabaster, AL, USA). Approximately 95–97% of LPA is lost when reconstituted without BSA [23] or suitable organic solvents. Therefore, stable lipids (i.e.*,*16:0 LPA) were dissolved in EtOH:H_2_O (1:1 v/v), and unstable and highly hygroscopic lipids (i.e.*,* 18:1 LPA, 18:1 LPC, 18:1–18:1 PA) were desiccated, then reconstituted in EtOH:H_2_O (1:1 v/v solution) to prepare 5 mM stock solutions. 18:1 S1P was dissolved in methanol, desiccated, and reconstituted in 0.4% BSA according to the manufacturer’s instructions.

### FSA configuration

**Fig. 2:**
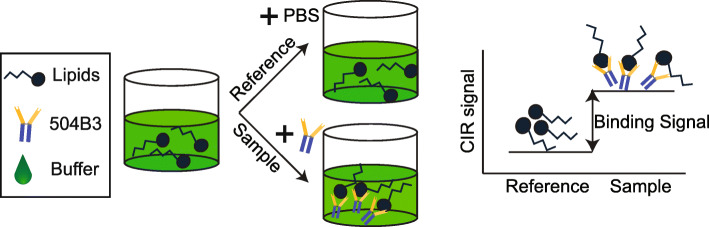
FSA configuration to analyze by CIR. Lipid solutions (six to seven different concentrations each) were divided into two aliquots: one with 504B3 antibody in PBS to form the binding *sample*; and one with PBS only to prepare an RI-matched non-binding *reference* solution. The binding signal is determined by RI change between the *reference* and the *sample*

The FSA configuration required refractive index (**RI**)-matched *sample* and *reference* solutions (Fig. 2). The binding affinity determinations were done in an end-point assay format [21]. A ligand dilution series (100, 20, 4, 0.8, 0.16, 0.032, and 0 nM) was prepared in 0.01% BSA / 0.002% EtOH/Phosphate buffered saline (**PBS**; ThermoFisher Scientific, Waltham, MA, USA) from an intermediate stock of 200 nM LPs in 0.01% BSA / 0.002% EtOH/PBS. Lipids were prepared in fatty acid-free BSA solutions to approximate the biological conditions of LPs. Then, each LP dilution was combined with PBS-only to create the *reference* solution, or 10 μg/ml of the 504B3 antibody in PBS to create the corresponding binding *sample* solution (Fig. 2). *Sample* and *reference* solutions were kept at RT in a shaker for approximately 1-h to reach equilibrium and then analyzed using the CIR. The final concentrations were 5 μg/ml of antibody and 0–50 nM of ligand in a final buffer composition of 0.005% BSA / 0.001% EtOH/PBS.

### The CIR

The details of the interferometer instrumentation have been described elsewhere [17, 19, 20]. Briefly, it consists of a diode laser, two mirrors, one glass capillary, and a CCD camera. Droplet trains of *sample-reference* pairs were maintained at a constant flow rate in the capillary using a droplet generator (Mitos Dropix; Dolomite Microfluidics, (Royston, North Hertfordshire, UK)) and a syringe pump (Harvard Apparatus, Holliston, MA, USA). Together, the interferometer, a syringe pump (at a flow rate of 10 μL/min), and a droplet generator comprise the CIR. The RI change between the binding *sample* and *reference* was measured as a positional shift in backscattered interference fringes produced from the interaction between an expanded beam profile of the laser and a capillary filled with *sample-reference* solutions [24]. The shift of the backscattered fringe patterns, which is equivalent to molecular binding, was quantified using fast Fourier transform of selected fringes captured in a CCD array. The assay was measured sequentially, as *reference*, then *sample* for each concentration, as described previously [19]. The concentration dependent signal was plotted using a non-linear regression, one-site specific binding (Y=B_max_*X/(K_D_ + X) + D; where D is the offset value) isotherm fit in GraphPad Prism Version 8 to determine the equilibrium dissociation constants (K_D_s).

## Results

**Fig. 3:**
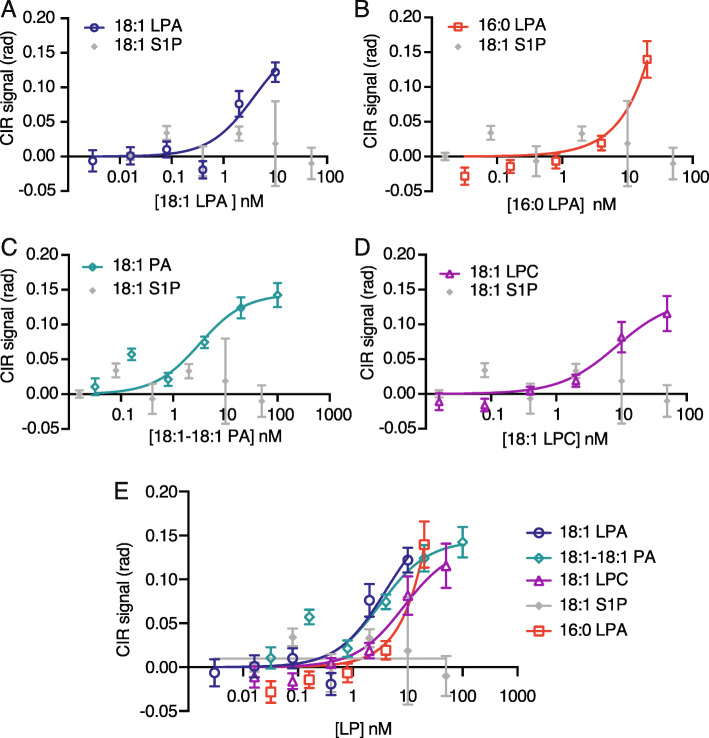
CIR signals identified binding of 504B3 with 4 different phospholipid forms. **a-e** CIR signal was plotted against the ligand concentrations to determine the binding constant (K_D_) for 504B3 anti-LPA antibody against five lysophospholipid forms. Binding curves for each ligand ((**a**) 18:1 LPA, (**b**) 16:0 LPA, (**c**)18:1 PA and (**d**)18:1 LPC and (**e**) all ligands) were plotted with the negative control S1P (no signal). Each data point represents multiple binding isotherms (experimental replicates) (18:1 LPA, *N* = 4; 16:0 LPA, *N* = 4; 18:1–18:1 PA, *N* = 2; 18:1 LPC, *N* = 4, and 18:1 S1P, *N* = 2), each with six to seven technical replicates

FSA-CIR was used to reevaluate the equilibrium binding affinity (K_D_) of the anti-LPA mAb (504B3) against five phospholipids (Fig. 1; 18:1 LPA, 16:0 LPA, 18:1 LPC, 18:1 S1P, and 18:1–18:1 PA). The *sample* and *reference* solutions were prepared by mixing a ligand dilution series (0–100 nM) with an equivalent volume of the 504B3 solution (*sample*; 10 μg/ml dissolved in PBS, pH 7.4) or PBS only (*reference*) (Fig. 2). Use of the FSA-CIR methodology showed a robust concentration dependent ΔRI signal that almost reached saturation for all ligands, except 16:0 LPA. The FSA signal (ΔRI; refractive index difference between *sample* and *reference*) was plotted against ligand concentrations and showed nanomolar binding affinities of 504B3 to not only 18:1 LPA (K_D_ ≈ 3.73 ± 2.8 nM), but also to 18:1 LPC (K_D_ ≈ 8.5 ± 2.6 nM) and 18:1–18:1 PA (K_D_ ≈ 3.3 ± 2.7 nM), with weaker affinity for 16:0 LPA (Fig. 3; Table 1). No specific binding signal was observed for 18:1 S1P.

**Table 1:** Binding constants (K_D_) determined for 5 different phospholipid forms against 504B3

Lysophospholipid	K_D_ from FSA ± SD (95% CI)	B_max_ (95% CI)	R^2^
18:1 LPA	3.7 ± 2.8 nM (2.1 to 9.0)	0.17 (0.14 to 0.25)	0.83
18:1 PA	3.3 ± 2.7 nM (1.5 to 6.4)	0.15 (0.12 to 0.17)	0.81
18:1 LPC	8.5 ± 2.6 nM (5.3 to 13.5)	0.14 (0.12 to 0.16)	0.90
16:0 LPA	> 30 nM (undetermined^a^)	0.39 (undetermined^a^)	0.94
18:1 S1P	~ 0	–	–

^a^Output as “very wide” in GraphPad Prism version 8

## Discussion

A strong affinity of 504B3 to 18:1 LPA was detected, contrasting with 16:0 LPA, which is consistent with previously defined GPCR binding affinities that showed weaker binding to LPA species with shorter hydrocarbon tails [25]. Notably, prior analyses showed comparable binding between LPA_1_ and both 18:1 LPA and 16:0 LPA [19]. These data suggest that the observed reduced binding affinities for 16:0 LPA may have physiological significance intrinsic to molecular interactions required for binding, albeit with the caveat that antibodies and GPCRs are very different proteins, with different physiological locations: antibodies (IgGs) are in solution, whereas LPA_1_ and other GPCRs are membrane-bound, all of which complicate direct binding comparisons. However, 504B3-LPA binding showed similar affinities to those seen for LPA when assayed for 18:1–18:1 PA and 18:1 LPC. This high-affinity, off-target binding suggests that neither the head group (18:1 LPC), nor the presence of a 2nd unsaturated hydrocarbon chain (18:1–18:1 PA), significantly altered binding interactions of this antibody. Overall, 504B3 shows comparable binding to 18:1 species of LPC, PA, and LPA.

Importantly, endogenous plasma concentrations of LPC (100–300 μM) are 100-times the reported LPA concentrations (0.1–2 μM) [26–28], indicating that a vast majority of 504B3 and related antibodies used in vivo would likely be bound to LPC rather than LPA.

### Strengths and limitations of the study

FSA-CIR possesses a number of strengths compared to other technologies: 1) FSA-CIR measures binding in a free solution without a need for tethering or other forms of immobilization, thus better approximating conditions present in vivo between LPA and a binding protein; 2) FSA-CIR does not require molecular labels – e.g.*,* radioactivity, fluorescence, or epitopes – thus avoiding potential artifactual effects; and 3) it utilizes minute volumes, e.g.*,* nanoliter or even sub-nanoliter are sufficient for binding. Despite these strengths, FSA-CIR has its limitations: 1) it is relatively limited in throughput and is without automation; 2) it is not readily available to the research community and is not currently commercialized; and 3) it requires thorough cleaning of the microfluidic channel to avoid cross-sample contamination limiting its scalability. The application used here was limited in that it did not provide stochiometric assessments of LPA molecules bound to an antibody (although use of the two-site binding model did not result in reliable curve fitting based on R^2^ and other parameters), and not all ligand binding curves reached saturation, which we speculate could reflect interactions beyond antigen binding sites facilitated by lipid properties that, for example, lead to micelles at higher LPA concentrations [29].

## Conclusions

The anti-LPA antibody, 504B3, binds other lipids beyond LPA. The use of antibody-based therapies has produced multiple therapeutic successes, ranging from cancer to autoimmune and infectious diseases [30–33], with the majority targeting protein epitopes. By contrast, bioactive lipids present more challenging targets in that they have overlapping epitopes, are present within most if not all cell membranes (e.g.*,* LPA roles in vesicular curvature [34]), bind to proteins in biological fluids (e.g.*,* to albumin), and have dynamic biosynthesis and release (e.g.*,* with platelet degranulation [35, 36]). As a result, LPA-targeting antibodies face the difficult task of removing actively and continuously produced LPA within not only signaling pools but also larger pools of structural membranes, vesicles, and other contributing compartments (e.g.*,* platelet granules).

More practically, these results raise mechanistic questions about the use of this and related anti-LPA antibodies in basic and clinical research, including pre-clinical studies on spinal cord injury [8] and traumatic brain injury [9, 14] in view of their lack of specificity for LPA, and the possibility that other lipids not assessed in this study could further complicate interpretations. More broadly, other antibodies generated against lipids may benefit from binding analyses by FSA-CIR, particularly for studies of the brain that is composed primarily of lipids (by dry mass). The clinical utility of such strategies remains to be demonstrated; however, it is formally possible that shared epitopes on off-target molecules could nonetheless provide novel results with clinical potential, albeit through distinct mechanisms.

## Data Availability

All data generated or analyzed during this study are included in this published article.
